# Afferent Connections to the Rostrolateral Part of the Periaqueductal Gray: A Critical Region Influencing the Motivation Drive to Hunt and Forage

**DOI:** 10.1155/2009/612698

**Published:** 2009-03-19

**Authors:** Sandra Regina Mota-Ortiz, Marcia Harumi Sukikara, Luciano Freitas Felicio, Newton Sabino Canteras

**Affiliations:** ^1^Laboratory of Neurosciences, City University of Sao Paulo, UNICID, Rua Cesário Galeno 448, 03071-000 Sao Paulo, SP, Brazil; ^2^Department of Anatomy, Institute of Biomedical Sciences, University of Sao Paulo, Avenida Professor Lineu Prestes 2465, 05508-900 Sao Paulo, SP, Brazil; ^3^Department of Pathology, School of Veterinary Medicine and Zootechny, University of Sao Paulo, Avenida Professor Dr. Orlando Marques de Paiva 87, 05508-270 Sao Paulo, SP, Brazil

## Abstract

Previous studies have shown that a particular site in the periaqueductal gray (PAG), the rostrolateral PAG, influences the motivation drive to forage or hunt. To have a deeper understanding on the putative paths involved in the decision-making process between foraging, hunting, and other behavioral responses, in the present investigation, we carried out a systematic analysis of the neural inputs to the rostrolateral PAG (rlPAG), using Fluorogold as a retrograde tracer. According to the present findings, the rlPAG appears to be importantly driven by medial prefrontal cortical areas involved in controlling attention-related and decision-making processes. Moreover, the rlPAG also receives a wealth of information from different amygdalar, hypothalamic, and brainstem sites related to feeding, drinking, or hunting behavioral responses. Therefore, this unique combination of afferent connections puts the rlPAG in a privileged position to influence the motivation drive to choose whether hunting and foraging would be the most appropriate adaptive responses.

## 1. Introduction

Previous studies from our laboratory, examining the neural basis of morphine-induced inhibition of maternal behavior, brought up the suggestion of a rather unsuspected and integrative role of the periaqueductal gray (PAG) in influencing the selection of adaptive behavioral responses [1, 2].

Examining the neural basis underlying maternal behavior inhibition by low doses of morphine in morphine-experienced dams, we found that morphine treatment induces a behavioral switch from maternal to predatory behavior. Hence, morphine-challenged dams, tested in an environment containing both pups and roaches (which served as prey), clearly preferred hunting instead of nursing [2]. We have further shown that, under physiological conditions, there is a natural endogenous opioid tone that may be able to stimulate hunting in lactating dams [3].

The results of behavioral, neuronal immediate early gene activation, and lesion experiments indicate that a particular site in the PAG, at the level of the oculomotor nucleus, located in the outer half of the lateral column, and referred to as the rostrolateral PAG (rlPAG), should be responsible for this switching from maternal behavior to prey hunting in morphine-treated dams [2]. First, we showed that the rlPAG upregulates Fos expression in lactating rats acutely challenged with morphine [1], similar to what had been found for animals performing insect hunting [4]. Next, by testing morphine-treated dams in an environment containing pups and roaches, we were able to show that lesions of the rlPAG, but not other parts of the PAG, impaired predatory hunting and restored the maternal response [2]. These findings support the idea that this opioid sensitive PAG site is critical for influencing the motivation drive to hunt and forage; and should be a nodal part of a neural circuit involved in the decision-making process between hunting, foraging, and other behavioral responses.

To start unraveling this circuit, in the present study, we performed a comprehensive investigation on the rlPAG afferent connections, using the Fluorogold as retrograde tracer. A number of retrograde tract-tracing studies have investigated the afferent sources of inputs to the PAG, but they used a much less sensitive retrograde tracer (i.e., the retrograde transport of the horseradish peroxidase) and were based on large injection sites encompassing different PAG functional domains [5]. The retrograde tract-tracing method using Fluorogold as a tracer, and revealed by immunohistochemical procedures, is one of the most sensitive retrograde tract-tracing tools available [6], and yields relatively small injection sites, a feature particularly suitable for investigating the afferent connections of relatively small sites, such as the rlPAG, in the present case.

Overall, the present results support the idea that the rlPAG combines a unique set of inputs rendering this region particularly suitable for influencing the decision-making process between hunting, foraging, and other behavioral responses.

## 2. Materials and Methods

### 2.1. Animals

Subjects were adult female Wistar rats (*n* = 18) weighing 190–220 g and approximately 90 days of age at the beginning of the experiments. Food and water were available ad libitum to the animals in light-controlled (06:00 AM to 06:00 PM) and temperature-controlled (23–25°C) rooms. Conditions of animal housing and all experimental procedures were conducted under institutional guidelines of the Committee on Animals of the (Colégio Brasileiro de Experimentação Animal, Brazil) and the Committee on the Care and Use of Laboratory Animal Resources, National Research Council.

### 2.2. Retrograde Tracing Experiments

Animals were anesthetized with a mixture of ketamine (Vetaset; Fort Dodge Laboratory, Campinas, Brazil) and xylazine (Rompum, 1:2 v/v; 1 mL/kg body weight; Bayer; Sao Paulo, Brazil), and unilateral iontophoretic deposits of a 2% solution of Fluorogold (Fluorochrome Inc., Colo, USA) were placed stereotaxically into the rlPAG (2.9 mm rostral to the interaural line, 0.65 mm from the midline, and 4.5 mm ventral to the surface of the brain). Deposits were made over 5 minutes through a glass micropipette (tip diameter, 25 *μ*m) by applying a +3 *μ*A current, pulsed at 7-second intervals, with a constant-current source (Midgard Electronics, Wood Dale, Ill, USA, model CS3). After a survival time of 7–12 days, the animals were deeply anesthetized with sodium pentobarbital (65 mg/kg, IP) and perfused transcardially with a solution of 4.0% paraformaldehyde in 0.1 M phosphate buffer, pH 7.4; the brains were removed and left overnight in a solution of 20% sucrose in 0.1 M phosphate buffer at 4°C. The brains were then frozen, and five series of 30-*μ*m-thick sections were cut on a sliding microtome in the transverse (frontal) plane and collected from the caudal medulla through the rostral tip of the prefrontal cortex. One complete series was processed for immunohistochemistry with an antiserum directed against Fluorogold (Chemicon International, Calif, USA) at a dilution of 1:5000. The antigen-antibody complex was localized with a variation of the avidin-biotin complex system (ABC) [7], with a commercially available kit (ABC Elite Kit, Vector laboratories, Calif, USA). The sections were mounted on gelatin-coated slides and then treated with osmium tetroxide to enhance visibility of the reaction product. Slides were then dehydrated and cover slipped with DPX. An adjacent series was always stained with thionin to serve as reference for cytoarchitecture.

Sections were examined under a microscope with bright- and dark-field illumination. Fluorogold deposits in the injection sites, and the distribution of retrogradely labeled neurons, were plotted with the aid of a camera lucida onto maps prepared from adjacent thionin-stained sections. The distribution of retrograde labeling was transferred onto a reference atlas of the rat brain [8]. The figures were prepared using Adobe PhotoShop (v.5.5; Adobe Systems, San Jose, Calif, USA) for photomicrographs and Adobe Illustrator (v.10, Adobe Systems) for drawings.

## 3. Results

**Figure 1:**
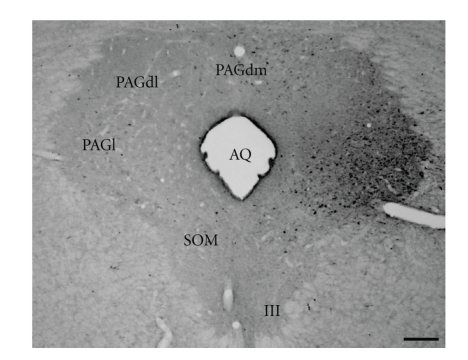
Brightfield photomicrograph illustrating the appearance of Fluorogold (FG) injection site in experiment PAGlFG15. Scale bar = 200 *μ*m.

**Figure 2:**
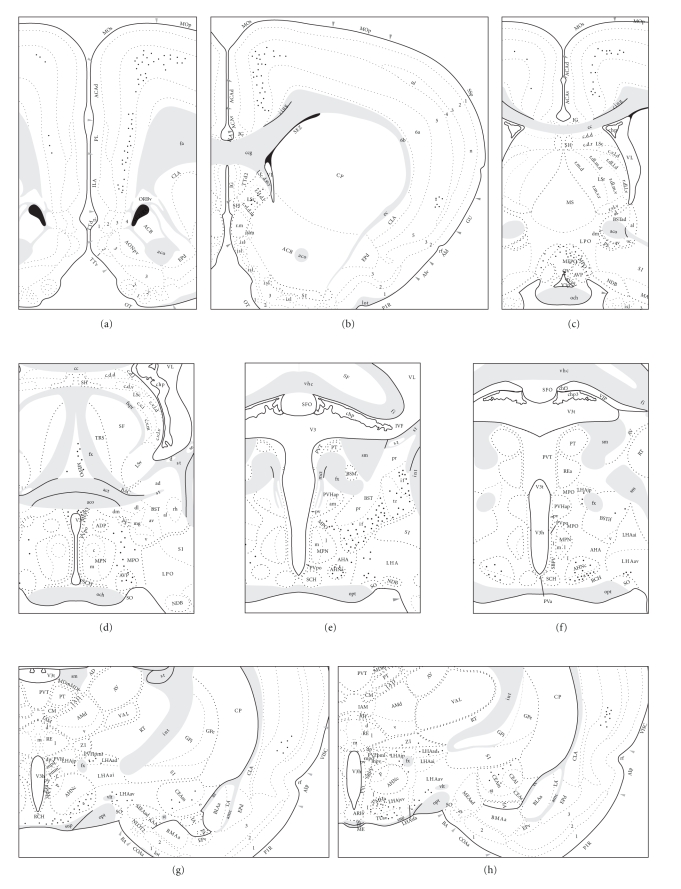

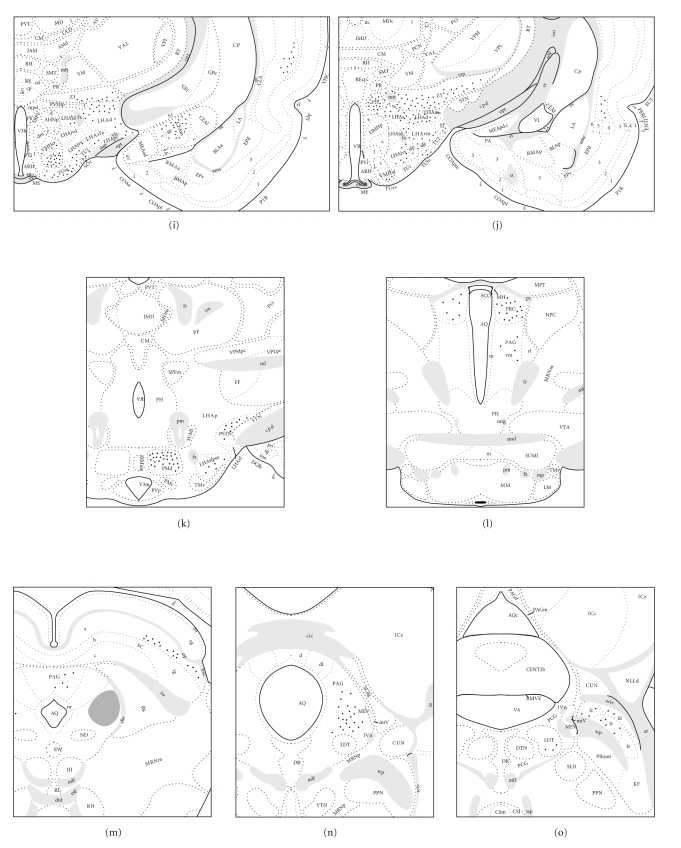
Inputs to the rlPAG. The distribution of retrogradely labeled neurons (black dots) in experiment PAGlFG15 plotted onto a series of standard drawings of the rat brain arranged from the rostral (a) to caudal (o) levels. The dark gray area indicates the FG injection site in this experiment. For abbreviations, see Supplementary Material available online at doi:10.1155/2009/612698.

The distribution of neurons projecting to the rlPAG region was examined by using Fluorogold. In five experiments, the deposit of the tracer appeared to be confined almost entirely to the rlPAG (located in the outer half of the lateral column at the levels of the oculomotor nucleus). The appearance of a representative Fluorogold injection site in the rlPAG is illustrated in Figure 1, and the distribution of retrogradely labeled neurons from this experiment is illustrated schematically in Figure 2. The results of this experiment are described in detail, because the injection site was virtually confined to the rlPAG. Furthermore, in this experiment, the pattern of retrograde labeling was representative of that one seen in each of the other experiments with deposits centered in the rlPAG. The following is a summary of regions that appear to send fibers to the rlPAG.

### 3.1. Telencephalon

Retrogradely labeled neurons were found in the isocortex, amygdala and in the septal region. No retrogradely labeled cells were found in the hippoccampal formation.

**Figure 3:**
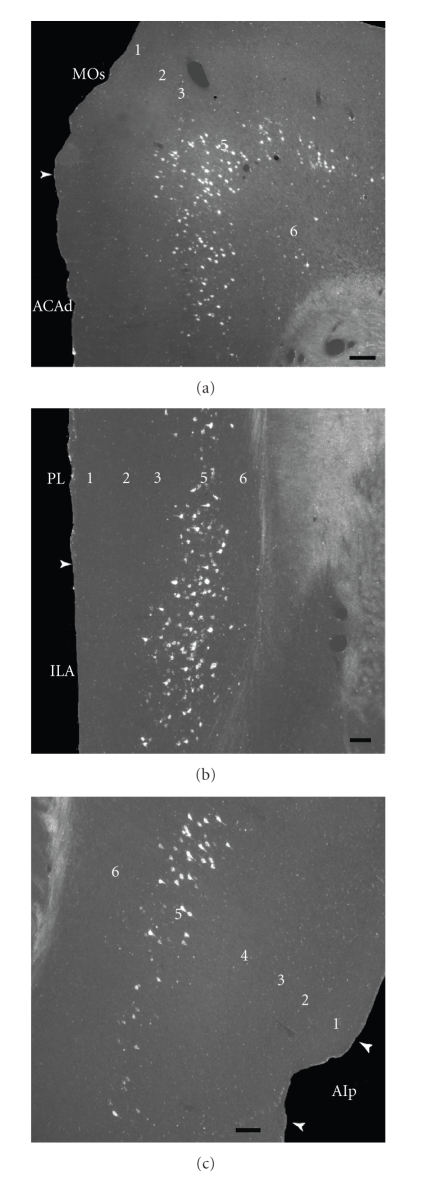
Dark field photomicrographs showing (a) the distribution of retrogradely labeled cells within the ipsilateral secondary motor area and the dorsal part of the anterior cingulated area; (b) the prelimbic and infralimbic areas; (c) and the visceral area. Scale bars = 200 *μ*m.

In the isocortex, a large number of retrogradely labeled neurons was observed in the prelimbic, infralimbic, anterior cingulate, and secondary motor areas (Figures 2(a), 2(b), 2(c) and 3(a), 3(b)), in addition to substantial labeling in the gustatory and visceral areas (Figures 2(b), 2(g), 2(h), and 2(i)). A few retrogradely labeled neurons were found in the posterior part of agranular insular area (Figure 3(c)) and primary motor, perirhinal, and ectorhinal areas. All of the cortical labeled cells were pyramidal neurons of the layer V.

**Figure 4:**
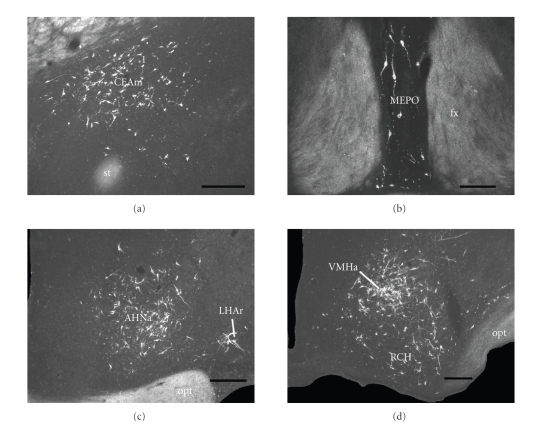
Dark field photomicrographs showing (a) the distribution of retrogradely labeled cells within the ipsilateral medial part of the central nucleus of the amygdala; (b) the median preoptic nucleus; (c) the anterior part of the anterior hypothalamic nucleus and the retinoceptive region of the lateral hypothalamic area; (d) and the anterior part of the ventromedial hypothalamic nucleus and the retrochiasmatic area. Scale bars = 200 *μ*m.

In the lateral septal nucleus, a relatively sparse number of marked cells were observed in the rostral part of the nucleus, distributed mainly through the dorsal region of the ventrolateral zone (Figures 2(b), 2(c)). In the septal region, we have also observed a substantial number of retrogradely marked neurons in the posterior division of the bed nuclei of the stria terminalis, particularly in the interfascicular nucleus, and also, to a lesser degree, in the transverse nucleus (Figures 2(e), 2(f)). In the amygdala, a large number of retrogradely labeled cells were found to be restricted to the medial part of the central amygdalar nucleus (Figures 2(g), 2(h), 2(i), and 4(a)).

### 3.2. Diencephalon

**Thalamus**

At the thalamus, a substantial retrograde labeling was found in the ventral part of the zona incerta (Figures 2(h), 2(i), 2(j)). No marked cells were observed in the dorsal thalamus.

**Hypothalamus**

In thepreoptic region, a substantial retrograde labeling was found in the median preoptic nucleus (Figures 2(c), 2(d), and 4(b)), in addition to a sparse number of marked cells in the anteroventral preoptic nucleus (Figures 2(c) and 2(d)).

At the anterior hypothalamic levels, the anterior part of the anterior hypothalamic nucleus presented a dense cluster of retrogradely labeled cells, which were distributed in the region that seems to overlap, at least partially, with a territory known to contain a large number of neurons expressing enkephalin [9] (Figures 2(e), 2(f), 2(g), and 4(c)). Moreover, at these levels, a substantial retrograde labeling was also found in the lateral hypothalamic area immediately dorsal to the optic tract and the supraoptic nucleus, which seems to correspond to a region densely targeted by the lateral component of retinohypothalamic tract [10] (Figures 2(f), 2(g), and 4(c)).

At tuberal levels, a dense number of retrogradely labeled cells was found in the anterior part of the ventromedial nucleus, in addition to a moderate number of marked cells in the retrochiasmatic area, the ventrolateral and central parts of the ventromedial nucleus, the tuberal nucleus, and rostral parts of the posterior hypothalamic nucleus (Figures 2(g), 2(h), 2(i), 2(j), and 4(d)). Furthermore, at these levels, we have found a large number of retrogradely labeled cells in the lateral hypothalamic area, distributed in the dorsal, suprafornical, justadorsomedial, and justaventromedial areas (Figures 2(g), 2(h), 2(i), and 2(j)).

At the mammilary levels, a large number of labeled neurons were found in the dorsal premammilary nucleus, mostly distributed in the dorsal part of the nucleus (Figure 2(k)). Finally, at these levels, we have found moderate retrograde labeling in the parasubthalamic nucleus, in addition to sparse labeling in the subfornical region of the lateral hypothalamic area (Figures 2(j) and 2(k)).

**Brainstem**

At mesodiencephalic levels, a large number of marked cells were found in the precommissural nucleus (Figure 2(l)). In the midbrain, at the injection site level, substantial retrograde labeling was found in the lateral part of the intermediate layer of the superior colliculus (Figure 2(m)). Additionally, at this level, a moderate number of retrogradely labeled neurons were also found in the PAG, which appeared to be distributed within the dorsomedial part (Figure 2(m)). Proceeding caudally, at the intermediate rostrocaudal levels of the dorsal raphe nucleus, a moderate number of marked cells was found in the ventrolateral part of the PAG (Figure 2(n)).

At rostral pontine levels, a few labeled cells were found in the laterodorsal tegmental nucleus, as well as in the central lateral, dorsal lateral, and ventral lateral parts of the parabrachial nucleus (Figure 2(o)).

It should be noted that, in the experiments with Fluorogold deposits centered in the rlPAG, retrograde labeling was mostly ipsilateral. However, some of the main sources of projections to this area, including the prefrontal cortex, retrochiasmatic area, anterior hypothalamic nucleus, ventromedial hypothalamic nucleus, lateral hypothalamic area, zona incerta, precommissural nucleus, and the PAG, also displayed conspicuous retrograde labeling contralateral to the injection site.

## 4. Discussion

The results of the present retrograde axonal tract-tracing study suggest that the rlPAG receive inputs from several widely distributed areas in the forebrain and, to a lesser extent, from the brainstem, as well. Prefrontal cortical areas represent the major telencephalic source of inputs to the rlPAG. In addition, clear telencephalic inputs appear to arise from the gustatory, visceral, and perirhinal cortical areas, as well as from the medial part of the central amygdalar nucleus and the interfascicular nucleus of the bed nuclei of the stria terminalis. In the diencephalon, massive inputs to the rlPAG arise from several hypothalamic sites, including the median preoptic nucleus, anterior hypothalamic nucleus, retrochiasmatic area, anterior, and ventrolateral parts of the ventromedial nucleus, dorsal premammillary nucleus (PMd), and several districts of the lateral hypothalamic area. In contrast, the rlPAG appears to receive inputs from considerably fewer brainstem sites, where significant retrograde labeling was found in other parts of the PAG (i.e., the dorsomedial and ventrolateral parts) and in the intermediate layers of the lateral part of the superior colliculus.

In agreement with previous anterograde tract-tracing studies, medial prefrontal cortical areas, including the infralimbic, prelimbic, anterior cingulated, and secondary motor areas, appear to represent one of the most important afferent sources of projections to the rlPAG [11–20]. In line with previous anterograde findings, we have also found that the rlPAG receives projections from visceral, gustatory, and perirhinal cortical areas [12–14, 17]. In the septal region, a substantial number of retrograde labeled cells were found in the interfascicular nucleus of the BST, a finding likewise supported by previous PHAL studies [21]. The present results also revealed that the medial part of the central nucleus of the amygdala is an important source of telencephalic inputs to the rlPAG. This finding has also been confirmed by means of PHAL anterograde tract-tracing method, which showed that the medial part of the central nucleus of the amygdala provides a substantial terminal field to the lateral PAG [22].

The present findings also revealed that the rlPAG is targeted by several hypothalamic districts. At preopotic levels, the rlPAG seems to receive substantial inputs from the median preoptic nucleus, in addition to somewhat sparse inputs from the anteroventral preoptic nucleus. Both of these projections have been confirmed by previous PHAL studies [23]. At anterior hypothalamic levels, a prominent group of retrogradely labeled cells was found in the anterior part of the anterior hypothalamic nucleus, in a region containing a characteristic cluster of enkephalinergic cells [9]. We were able to confirm this projection by means of PHAL deposits placed in the anterior part of the anterior hypothalamic nucleus, which yielded a distinct terminal field in the rlPAG [S. R. Mota-Ortiz and N. S. Canteras, personal observation]. In addition, at these levels, we have found that the rlPAG receives inputs from the retrochiasmatic area and a lateral hypothalamic region immediately adjacent to the optic tract and the supraoptic nucleus, which receives direct projections from the lateral part of the retinohypothalamic tract [10] and corresponds to the so-called retinoceptive region of the lateral hypothalamic area. Both the projections from the retrochiasmatic area and from the retinoceptive region of the lateral hypothalamic area to the rlPAG have been previously confirmed through PHAL experiments [24, N. S. Canteras, personal observation]. At tuberal levels, in agreement with the results of our retrograde transport experiments, previous evidence based on PHAL anterograde tract-tracing indicates that the rlPAG receives dense projections from the anterior part of the ventromedial nucleus and the posterior hypothalamic nucleus, in addition to somewhat weaker inputs from the ventrolateral part of the ventromedial nucleus and the adjacent tuberal nucleus [25, 26]. In addition, at these levels, we found that the rlPAG receives substantial inputs from the dorsal, suprafornical, justadorsomedial, and justaventromedial areas of the lateral hypothalamus, as well as immediately adjacent parts of the zona incerta. These projections have been confirmed in PHAL studies [27, M. Goto, personal observation]. Notably, this region of the lateral hypothalamus overlaps with the region of cells expressing melanin-concentrating hormone (MCH) and hypocretin/orexin [28]. Moreover, in agreement with previous PHAL studies [29], we have also found, in the lateral hypothalamic area, that the parasubthalamic nucleus represents another source of inputs to the rlPAG. Finally, at mammilary levels, we found a distinct projection mostly from the dorsal part of the PMd. The PMd represents one of the main hypothalamic sources of inputs to the PAG [5], and previous PHAL studies have shown that the dorsal and ventral parts of the nucleus provide a differential pattern of projection to the PAG, and confirmed that the dorsal part of the PMd provides a strikingly dense projection to the rlPAG [30].

The present results also revealed that a small number of brainstem sites appear to innervate the rlPAG. Evident retrograde labeling was found in the precommissural nucleus, the dorsomedial and ventrolateral parts of the PAG, and the lateral part of the intermediate layer of the superior colliculus. All of these projections have been confirmed through PHAL anterograde tract-tracing method [31, S. R. Mota-Ortiz and N. S. Canteras, personal observation] In addition, we have also found that the rlPAG receives relatively sparse projections from the laterodorsal tegmental nucleus and the lateral part of the parabrachial nucleus. Unfortunately, to our knowledge, these projections remain yet to be demonstrated with anterograde tracer studies.

### 4.1. Differential Inputs to Other PAG Parts: Comparison with the Afferent Connections to the Dorsolateral PAG (dlPAG)

As mentioned above, previous retrograde tract-tracing studies have investigated the afferent sources of inputs to the PAG, but they were based on large injection sites, providing just an overall picture of the afferent inputs to the PAG without differentiating among the particular PAG domains [5]. By determining the specific set of afferent inputs to the rlPAG, we have provided important information to distinguish this PAG site from adjacent different functional domains, such as the immediately adjacent dorsolateral PAG (dlPAG). In contrast to the rlPAG, the dlPAG upregulates Fos expression in response to both actual and contextual predatory threats [32–34], and seems to be responsible for organizing the expression of unconditioned and conditioned antipredatory responses [33]. As for the rlPAG, the dlPAG also receives substantial inputs from the anterior cingulate, prelimbic, and infralimbic areas [11, 12, 19, 20], but in sharp contrast to what has been found for the rlPAG, the visceral area appears to project only sparsely to the dlPAG [13]. Additionally, in contrast to the rlPAG, the dlPAG does not seem to receive substantial inputs from the amygdala, and seems to be densely innervated by medial hypothalamic sites involved in processing predatory cues, including the posterior part of the anterior hypothalamic nucleus, the dorsomedial part of the ventromedial nucleus and the dorsal premammillary nucleus [25, 35, 36]. Notably, the projection from the dorsal premammillary nucleus to the dlPAG arises chiefly from the ventrolateral part of the nucleus, which is the hypothalamic site most responsive to the predator or its cues [30]. In the zona incerta, differently from what we have just found for the rlPAG, the dlPAG seems to receive dense projections from the rostral zona incerta, also referred to as the incertohypothalamic area [37]. The superior colliculus also has a differential pattern of projection to the PAG, where the lateral part of the intermediate layer projects to the rlPAG, as presently shown, and the medial part of the intermediate and deep layers target the dorsal PAG [38]. Notably, the medial part of the intermediate and deep layers of the superior colliculus respond to visual-threatening stimuli, such as suddenly expanding shadows in the upper visual field (which look like an approaching predator), and, via a projection to the dlPAG, may exert a marked influence on the control of defensive responses [38].

The evidence just discussed supports the idea that different functional PAG domains are likely to have particular sets of afferent inputs, and next, we will provide a discussion on how the diverse inputs to the rlPAG may be related to its function on hunting and foraging behavior.

### 4.2. Functional Considerations

As commented in the Introduction, the rlPAG is an opioid sensitive site, which is critical for influencing the motivation drive to hunt and forage, and is likely to be part of a neural circuit involved in the decision-making process between hunting, foraging, and other behavioral responses. The present findings help to reveal this complex network, and reinforce the rlPAG's nodal role in integrating a wealth of different kinds of information likely to influence foraging or hunting activity.

One of the main findings of the present investigation is that the rlPAG appears to be particularly driven by inputs from medial prefrontal cortical areas. In the present context, it is noteworthy that, in the prefrontal cortex, the anterior cingulate, the prelimbic, and the infralimbic cortices have been associated with diverse emotional, cognitive, and mnemonic functions that underlie attentional and decision-making processes [39, 40]. In addition to the prefrontal cortex, the rlPAG also seems to be influenced by the septohippocampal system via a projection from the enkephalinergic region of the anterior hypothalamic nucleus [36]. Of particular relevance in the present account, the septohippocampal system has been shown to have a role in prioritizing the temporal order of motivated responses, which, in the present case, seems to utilize an enkephalinergic pathway. Here, it is noteworthy to recall that the opioidergic influence on the rlPAG has been shown to control the selection of adaptive behavioral responses, switching from maternal to hunting behavior [1].

As commented in the Introduction, the rlPAG upregulated Fos expression in animals performing insect hunting, and in the present study, we have shown that the rlPAG integrates inputs from a number of neural sites related to the circuitry underlying predatory hunting. Thus, we have presently found that the medial part of the central amygdalar nucleus provides a significant projection to the rlPAG. The medial part of the central amygdalar nucleus is part of a distinct amygdalar circuit comprising; the anterior part of the cortical nucleus, the anterior part of the basomedial nucleus and the posterior part of the basolateral nucleus, which has been shown to be mobilized during insect predation and seems to be particularly involved in processing visceral, gustatory and olfactory information [41–44]. The central nucleus—the main output way station of this amygdalar circuit—has been shown to be involved in controlling feeding behavior [45, 46]. In particular, the nucleus appears to integrate food hedonic values [47, 48] and to influence searching and consumption of palatable food through a pathway involving opioidergic neurotransmission [48, 49]. In the context of feeding behavior, it is also particularly relevant to point out that, according to the present findings, the rlPAG is also in a position to integrate visceral and gustatory information from the visceral and gustatory cortical areas, and to a lesser degree, from the parabrachial area [50].

The rlPAG also seems to be innervated by lateral hypothalamic regions activated during predatory hunting, namely, the parasubthalamic nucleus and the region containing cells expressing MCH and hypocretin/orexin [44]. The parasubthalamic nucleus is also targeted by the medial part of the central amygdalar nucleus, and provides inputs to hindbrain control regions involved in modulating digestive and metabolic responses occurring in both cephalic and consummatory phases of feeding behavior [29]. The lateral hypothalamic region containing cells expressing MCH and hypocretin/orexin appears to be involved in controlling arousal and exploratory activity related to feeding behavior [27, 51, 52]. In fact, this lateral hypothalamic region may also represent an important interface for the hypothalamic periventricular sites involved in the control of homeostatic feeding [53]. Moreover, according to the present findings, the rlPAG also receives direct inputs from the retrochiasmatic region, which has also been included in the hypothalamic circuit controlling feeding [54].

We have presently shown that the rlPAG is also targeted by the lateral part of the intermediate layer of the superior colliculus, which we have also previously shown to be mobilized during insect hunting [55]. In fact, cells in this collicular region seem to respond to moving objects in the temporal visual field, and we have found that animals bearing lesions in this collicular region, besides failing to orient themselves toward the moving prey, were also clearly less motivated to pursue the roaches [I. C. Furigo and S. R. Mota-Ortiz, personal observation] perhaps reflecting the functional role of the superior colliculus—rlPAG pathway.

The rlPAG is also significantly targeted by hypothalamic districts that do not seem to be directly involved in controlling feeding or hunting behavior, namely, the median preoptic nucleus, the retinoceptive region of the lateral hypothalamic, area and the dorsal premammillary nucleus. The median preoptic nucleus is classically known to respond to plasma osmolarity and influence drinking behavior [23], and this pathway to the rlPAG may be thought of as having some role in the search of water supplies. Conversely, the projection from the retinoceptive region of the lateral hypothalamic area may bring the information regarding the environmental luminescence, which may be thought of as having a direct impact on foraging activity [N. S. Canteras, personal observation]. Finally, the dorsal part of the dorsal premammillary nucleus provides an important projection to the rlPAG. Unfortunately, to the best of our knowledge, the potential roles of this path remain yet to be determined.

Here, it is important to consider that functional studies using electrical or chemical stimulation have suggested a role for the lateral PAG in defensive responses. In the rat, chemical stimulation with kainic acid (KA) in the region of the rlPAG produced strong backward defense [56], a kind of response that would be expected when the animal is exposed to a predator. However, this finding is hard to conciliate with the fact that rlPAG does not seem to be activated in response to predator exposure [32], as would be expected. In reality, the effects of KA injection in this region should be taken very cautiously since the immediately adjacent dlPAG presents a much heavier binding to KA [57] when compared to the rlPAG. Actually, the dlPAG appears as the PAG site presenting the largest activation to predator exposure [32], and, as previously discussed, its hodological pattern is fully compatible with its role in antipredatory defense.

In addition, the concept of a distinct functional column for the entire lateral PAG should also be revised. In fact, the role here assigned to the rlPAG does not seem to apply to the caudal half of the lateral PAG, since lesions encroaching upon this latter PAG region do not seem to affect the motivational drive to hunt [2, M. H. Sukikara and S. R. Mota-Ortiz, personal observation]. Conversely, there is a prominent Fos upregulation in the caudal, but not in the rostral, lateral PAG in response to predator exposure [32, 33].

Overall, the present findings support the idea that the rlPAG has a central role in a complex network controlling the decision-making process between hunting, foraging, and other behavioral responses. On one hand, the rlPAG appears to be importantly driven by medial prefrontal cortical areas involved in controlling attentional and decision-making processes. On the other hand, the rlPAG receives a wealth of information from different neural sites related to feeding, drinking, or hunting behavioral responses. Therefore, this unique combination of afferent connections puts the rlPAG in a privileged position to influence the motivation drive to choose whether hunting and foraging are the most appropriate adaptive responses.

## Supplementary Material

The supplementary material contains a list of abbreviations mentioned in the article.
