# Supplementary material for: Afferent Connections to the Rostrolateral Part of the Periaqueductal Gray: A Critical Region Influencing the Motivation Drive to Hunt and Forage
Source: Neural Plast. 2009 Mar 19;2009:612698. doi: 10.1155/2009/612698 (PMC2657915; doi:10.1155/2009/612698)
Supplement: Supplementary file 1 — The supplementary material contains a list of abbreviations mentioned in the article. [file 612698.f1.doc]

**LIST OF ABBREVIATIONS**

AAA: Anterior amygdalar area

ab: Angular bundle

ACAd: Anterior cingulate area, dorsal part

ACAv: Anterior cingulate area, ventral part

ACB: Nucleus accumbens

aco: Anterior commissure, olfactory limb

act: Anterior commissure, temporal limb

AD: Anterodorsal nucleus

ADP: Anterodorsal preoptic nucleus

AHA: Anterior hypothalamic area

AHN: Anterior hypothalamic nucleus

AHNa: Anterior hypothalamic nucleus, anterior part

AHNc: Anterior hypothalamic nucleus, central part

AHNd: Anterior hypothalamic nucleus, dorsal part

AHNp: Anterior hypothalamic nucleus, posterior part

AId: Agranular insular area, dorsal part

AIv: Agranular insular area, ventral part

alv: Alveus

AM: Anteromedial nucleus thalamus

amc: Amygdalar capsule

AMd: Anteromedial nucleus thalamus, dorsal part

AMv: Anteromedial nucleus thalamus, ventral part

AONpv: Anterior olfactory nucleus, posteroventral part

APN: Anterior pretectal nucleus

AQ: Cerebral aqueduct

ARH: Arcuate nucleus hypothalamus

AV: Anteroventral nucleus thalamus

AVP: Anteroventral preoptic nucleus

AVPV: Anteroventral periventricular nucleus

BA: Bed nucleus accessory olfactory tract

BAC: Bed nucleus anterior comissure

BLA: Basolateral nucleus amygdala

BLAa: Basolateral nucleus amygdala, anterior part

BLAp: Basolateral nucleus amygdala, posterior part

BMAa: Basomedial nucleus amygdala, anterior part

BMAp: Basomedial nucleus amygdala, posterior part

bsc: Brachium of the superior colliculus

BSM: Bed nucleus stria medullaris

BST: Bed nuclei of the stria terminalis

BSTal: Bed nuclei of the stria terminalis, anterior division, anterolateral

area

BSTav: Bed nuclei of the stria terminalis, anterior division, anteroventral

area

BSTam: Bed nuclei stria terminalis, anterior division, anteromedial

area

BSTdm: Bed nuclei of the stria terminalis, anterior division, dorsomedial

nucleus

BSTdl: Bed nuclei of the stria terminalis, anterior division, dorsolateral nucleus

BSTfu: Bed nuclei of the stria terminalis, anterior division, fusiform

nucleus

BSTif: Bed nuclei of the stria terminalis, posterior division, interfascicular

nucleus

BSTju: Bed nuclei of the stria terminalis, anterior division, juxtacapsular

nucleus

BSTmg: Bed nuclei of the stria terminalis, anterior division, magnocellular

nucleus

BSTov: Bed nuclei of the stria terminalis, anterior division, oval

nucleus

BSTpr: Bed nuclei of the stria terminalis, posterior division, principal

nucleus

BSTrh: Bed nuclei of the stria terminalis, anterior division, rhomboid

nucleus

BSTtr: Bed nuclei of the stria terminalis, posterior division,transverse nucleus

BSTv: Bed nuclei of the stria terminalis, anterior division, ventral

nucleus

Cc: Corpus callosum

ccg: Corpus callosum, genu

CEA: Central nucleus amygdala

CEAc: Central nucleus amygdala, capsular part

CEAl: Central nucleus amygdala, lateral part

CEAm: Central nucleus amygdala, medial part

chp: Choroid plexus

cic: Inferior colliculus commissure

cing: Cingulum bundle

CL: Central lateral nucleus thalamus

CLA: Claustrum

CLI: Central linear nucleus raphe

CM: Central medial nucleus thalamus

COA: Cortical amygdalar nucleus

COAa: Cortical amygdalar nucleus, anterior part

COApl: Cortical nucleus amygdala, posterior part, lateral zone

COApm: Cortical nucleus amygdala, posterior part, medial zone

COM: Commissural nucleus, periaqueductal gray

CP: Caudoputamen

cpd: Cerebral peduncle

csc: Commissure superior colliculus

CUN: Cuneiform nucleus

DMH: Dorsomedial hypothalamic nucleus

DMHa: Dorsomedial hypothalamic nucleus, anterior part

DMHp: Dorsomedial nucleus hypothalamus, posterior part

DMHv: Dorsomedial hypothalamic nucleus, ventral part

DR: Dorsal nucleus raphe´

Dtd: Dorsal tegmental decussation

DTN: Dorsal tegmental nucleus

ec: External capsule

em: External medullary lamina thalamus

ENTm1-6: Entorhinal area, medial part, layers 1-6

ENTmv: Entorhinal area, medial part, ventral zone

EPd: Endopiriform nucleus, dorsal part

EPv: Endopiriform nucleus, ventral part

EW: Edinger-Westphal nucleus

fa: Corpus callosum, anterior forceps

FF: Fields of Forel

fi: Fimbria

fr: Fasciculus retroflexus

FS: Fundus of the striatum

fx: Columns of the fornix

fxpr: Precommissural fornix

GPe: Globus pallidus, external segment

GPi: Globus pallidus, internal segment

GU: Gustatory area

hf: Hippocampal fissure

IA: Intercalated nuclei amygdala

IAD: Interanterodorsal nucleus thalamus

IAM: Interanteromedial nucleus thalamus

IC: Inferior colliculus

IF: Interfascicular nucleus raphe´

IG: Indusium griseum

IGL: Lateral geniculate complex, intergeniculate leaflet

III: Oculomotor nucleus

ILA: Infralimbic cortical area

IMD: Intermediodorsal nucleus thalamus

INC: Interstitial nucleus of Cajal

int: Internal capsule

IPN: Interpeduncular nucleus

IPNc: Interpeduncular nucleus, central subnucleus

IPNlr: Interpeduncular nucleus, lateral subnucleus, rostral part

IPNr: Interpeduncular nucleus, rostral subnucleus

IV: Trochlear nucleus

IVn: Trochlear nerve

LA: Lateral amygdalar nucleus

LC: Locus ceruleus

LD: Lateral dorsal nucleus thalamus

LDT: Laterodorsal tegmental nucleus

LGd: Lateral geniculate complex, dorsal part

LGvl: Lateral geniculate complex, ventral part, lateral zone

LGvm: Lateral geniculate complex, ventral part, medial zone

LH: Lateral habenula

LHA: Lateral hypothalamic area

LHAad: Lateral hypothalamic area, anterior region, dorsal zone

LHAai: Lateral hypothalamic area, anterior region, intermediate

zone

LHAav: Lateral hypothalamic area, anterior region, ventral zone

LHAd: Lateral hypothalamic area, dorsal region

LHAjd: Lateral hypothalamic area, juxtadorsomedial region

LHAjp: Lateral hypothalamic area, juxtaparaventricular region

LHAjv: Lateral hypothalamic area, juxtaventromedial region

LHAjvd: Lateral hypothalamic area, juxtaventromedial region, dorsal

Zone

LHAjvv: Lateral hypothalamic area, juxtaventromedial region, ventral

zone

LHAm: Lateral hypothalamic area, magnocellular nucleus

LHAp: Lateral hypothalamic area, posterior region

LHApc: Lateral hypothalamic area, parvicellular region

LHAs: Lateral hypothalamic area, suprafornical region

LHAsf: Lateral hypothalamic area, subfornical region

LHAvl: Lateral hypothalamic area, ventral region, lateral zone

LHAvm: Lateral hypothalamic area, ventral region, medial zone

LM lateral: Mammillary nucleus

lot: Lateral olfactory tract

LP: Lateral posterior nucleus thalamus

LPO: Lateral preoptic area

LS: Lateral septal nucleus

LSc: Lateral septal nucleus, caudal part

LSc.d: Lateral septal nucleus, caudal part, dorsal zone

LSc.v: Lateral septal nucleus, caudal part, ventral zone

LSc.v.l: Lateral septal nucleus, caudal part, ventral zone, lateral

region

LSc.v.l.d: Lateral septal nucleus, caudal part, ventral zone, lateral

region, dorsal domain

LSc.v.l.v: Lateral septal nucleus, caudal part, ventral zone, lateral

region, ventral domain

LSc.v.m.d: Lateral septal nucleus, caudal part, ventral zone, medial

region, dorsal domain

LSc.v.m.v: Lateral septal nucleus, caudal part, ventral zone, medial

region, ventral domain

LSr: Lateral septal nucleus, rostral part

LSr.dl: Lateral septal nucleus, rostral part, dorsolateral zone

LSr.dl.l: Lateral septal nucleus, rostral part, dorsolateral zone, lateral

region

LSr.dl.l.d: Lateral septal nucleus, rostral part, dorsolateral zone, lateral

region, dorsal domain

LSr.dl.l.v: Lateral septal nucleus, rostral part, dorsolateral zone, lateral

region, ventral domain

LSr.dl.m: Lateral septal nucleus, rostral part, dorsolateral zone, medial

region

LSr.dl.m.d: Lateral septal nucleus, rostral part, dorsolateral zone, medial

region, dorsal domain

LSr.dl.m.v: Lateral septal nucleus, rostral part, dorsolateral zone, medial

region, ventral domain

LSr.m: Lateral septal nucleus, rostral part, medial zone

LSr.m.d: Lateral septal nucleus, rostral part, medial zone, dorsal

region

LSr.m.v.c: Lateral septal nucleus, rostral part, medial zone, ventral

region, caudal domain

LSr.m.v.r: Lateral septal nucleus, rostral part, medial zone, ventral

region, rostral domain

LSr.vl.d.l: Lateral septal nucleus, rostral part, ventrolateral zone,

dorsal region, lateral domain

LSr.vl.d.m: Lateral septal nucleus, rostral part, ventrolateral zone,

dorsal region, medial domain

LSr.vl.v: Lateral septal nucleus, rostral part, ventrolateral zone,

ventral region

LSv: Lateral septal nucleus, ventral part

MA: Magnocellular preoptic nucleus

MD: Mediodorsal nucleus thalamus

MDc: Mediodorsal nucleus thalamus, central part

MDl: Mediodorsal nucleus thalamus, lateral part

MDm: Mediodorsal nucleus thalamus, medial part

MEA: Medial nucleus amygdala

MEAad: Medial amygdalar nucleus, anterodorsal part

MEAav: Medial amygdalar nucleus, anteroventral part

MEPO: Median preoptic nucleus

MEV: Midbrain nucleus of the trigeminal

MGm: Medial geniculate complex, medial part

MGv: Medial geniculate complex, ventral part

MH: Medial habenula

ml: Medial lemniscus

MM: Medial mammillary nucleus, body

MMme: Medial mammillary nucleus, median part

MOs: Secondary motor area

MOp: Primary motor area

moV: Motor root of the trigeminal nerve

mp: Mammillary peduncle

MPN: Medial preoptic nucleus

MPO: Medial preoptic area

MPT: Medial pretectal area

MRN: Midbrain reticular nucleus

MRNp: Midbrain reticular nucleus, parvicellular part

MS: Medial septal nucleus

mtt: Mammillothalamic tract

NB: Nucleus brachium inferior colliculus

ND: Nucleus of Darkschewitsch

NDB: Diagonal band nucleus

NLL: Nucleus of the lateral lemniscus

NPC: Nucleus of the posterior commissure

och: Optic chiasm

OP: Olivary pretectal nucleus

opt: Optic tract

ORBv: Orbital area, ventral part

OT: Olfactory tubercle

PA: Posterior nucleus amygdala

PAA: Piriform-amygdalar area

PAG: Periaqueductal gray

PAGd: Periaqueductal gray, dorsal division

PAGdl: Periaqueductal gray, dorsolateral division

PAGl: Periaqueductal gray, lateral division

PAGm: Periaqueductal gray, medial division

PAGrl: Periaqueductal gray, rostrolateral division

PAGrm: Periaqueductal gray, rostromedial division

PAGvl: Periaqueductal gray, ventrolateral division

PAR1-6: Parasubiculum, layers 1-6

PARN: Parvicellular reticular nucleus

PBl: Parabrachial nucleus, lateral part

PBlc: Parabrachial nucleus, central lateral part

PBlv: Parabrachial nucleus, ventral lateral part

PBmm: Parabrachial nucleus, medial medial part

pc: Posterior commissure

PCG: Pontine central gray

PCN: Paracentral nucleus thalamus

PF: Parafascicular nucleus

PH: Posterior hypothalamic nucleus

PIR: Piriform area

PL: Prelimbic cortical area

pm: Principal mammillary tract

PMD: Dorsal premammillary nucleus

PMDd: Dorsal premammillary nucleus, dorsal region

PMDv: Dorsal premammillary nucleus, ventral region

PMv: Ventral premammillary nucleus

PO: Posterior complex thalamus

PP: Peripeduncular nucleus

PPN: Pedunculopontine nucleus

PR: Perireuniens nucleus

PRC: Precommissural nucleus, periaqueductal gray

PRE1-6: Presubiculum, layers 1-6

PRNc: Pontine reticular nucleus, caudal part

PRNr: Pontine reticular nucleus, rostral part

PS: Parastrial nucleus

PSCH: Suprachiasmatic preoptic nucleus

PST: Preparasubthalamic nucleus

PSTN: Parasubthalamic nucleus of the lateral hypothalamic area

PSV: Principal sensory nucleus of the trigeminal

PT: Paratenial nucleus

PVa: Periventricular hypothalamic nucleus, anterior part

PVH: Paraventricular hypothalamic nucleus

PVHap: Paraventricular hypothalamic nucleus, anterior parvicellular

part

PVHf: Paraventricular nucleus hypothalamus, forniceal part

PVHlp: Paraventricular hypothalamic nucleus, lateral parvicellular

part

PVHmpd: Paraventricular hypothalamic nucleus, medial parvicellular

part, dorsal zone

PVHpml: Paraventricular hypothalamic nucleus, posterior magnocellular

part, lateral zone

PVHpv: Paraventricular hypothalamic nucleus, periventricular

part

PVi: Periventricular hypothalamic nucleus, intermediate part

PVp: Periventricular hypothalamic nucleus, posterior part

PVT: Paraventricular thalamic nucleus

Py: Pyramid

RCH: Retrochiasmatic area, lateral hypothalamic area

RE: Nucleus reuniens

REd: Nucleus reuniens, rostral division, dorsal part

REm: Nucleus reuniens, rostral division, median part

REv: Nucleus reuniens, rostral division, ventral part

RH: Rhomboid nucleus

RL: Rostral linear nucleus raphe

RM: Nucleus raphe´ magnus

RN: Red nucleus

RPO: Nucleus raphe´ pontis

RR: Midbrain reticular nucleus, retrorubral area

RT: Reticular nucleus thalamus

rust: Rubrospinal tract

SBPV: Subparaventricular zone hypothalamus

SC: Superior colliculus

SCdg: Superior colliculus, deep gray layer

SCdw: Superior colliculus, deep white layer

SCH: Suprachiasmatic nucleus

SCig: Superior colliculus, intermediate gray layer

SCiw: Superior colliculus, intermediate white layer

SCop: Superior colliculus, optic layer

scp: Superior cerebellar peduncle

SCsg: Superior colliculus, superficial gray layer

sctv: Ventral spinocerebellar tract

SCzo: Superior colliculus, zonal layer

SEZ: Subependymal zone

SF: Septofimbrial nucleus

SFO: Subfornical nucleus

SGN: Suprageniculate nucleus

SH: Septohippocampal nucleus

SI: Substantia innominata

SLC: Subceruleus nucleus

sm: Stria medullaris

smd: Supramammillary decussation

SMT: Submedial nucleus thalamus

SNc: Substantia nigra, compact part

SNr: Substantia nigra, reticular part

SO: Supraoptic nucleus

SPFm: Subparafascicular nucleus thalamus, magnocellular part

SPFpl: Subparafascicular nucleus thalamus, parvicellular part,

lateral division

SPFpm: Subparafascicular nucleus thalamus, parvicellular part,

medial division

SSp: Primary somatosensory area

st: Stria terminalis

STN: Subthalamic nucleus

SUBv: Subiculum, ventral part

SUBv-sp: Subiculum, ventral part, pyramidal layer

SUMl: Supramammillary nucleus, lateral part

SUMm: Supramammillary nucleus, medial part

sup: Supraoptic commissures

SUT: Supratrigeminal nucleus

SUV: Superior vestibular nucleus

TMv: Tuberomammillary nucleus, ventral part

TRS: Triangular nucleus septum

TR: Postpiriform transition area

tsp: Tectospinal pathway

TTd: Tenia tecta, dorsal part

TTv: Tenia tecta, ventral part

TU: Tuberal nucleus

TUi: Tuberal nucleus, intermediate part

TUl: Tuberal nucleus, lateral part

TUsv: Tuberal nucleus, subventricular part

TUte: Tuberal nucleus, terete subnucleus

V3: Third ventricle

V4: Fourth ventricle

VAL: Ventral anterior-lateral complex thalamus

vhc: Ventral hippocampal commissure

VISC: Visceral area

VL: Lateral ventricle

vlt: Ventrolateral hypothalamic tract

VM: Ventral medial nucleus thalamus

Vma: Motor nucleus of the trigeminal, magnocellular part

VMH: Ventromedial nucleus hypothalamus

VMHa: Ventromedial nucleus hypothalamus, anterior part

VMHc: Ventromedial nucleus hypothalamus, central part

VMHdm: Ventromedial nucleus hypothalamus, dorsomedial part

VMHvl: Ventromedial nucleus hypothalamus, ventrolateral part

VPL: Ventral posterolateral nucleus thalamus

VPLpc: Ventral posterolateral nucleus thalamus, parvicellular

part

VPM: Ventral posteromedial nucleus thalamus

VPMpc: Ventral posteromedial nucleus thalamus, parvicellular

part

VTA: Ventral tegmental area

vtd: Ventral tegmental decussation

VTN: Ventral tegmental nucleus

ZI: Zona incerta
